# Posterior mediastinal arteriovenous malformation: A rare catch

**DOI:** 10.34172/jcvtr.2021.11

**Published:** 2021-01-28

**Authors:** Pavneet Kohli, Prasanth Penumadu, Sagnik Ray, Rajesh Nachiappa Ganesh

**Affiliations:** ^1^Department of Surgical Oncology, JIPMER, Puducherry, India; ^2^Department of Pathology, JIPMER, Puducherry, India

**Keywords:** Posterior Mediastinal Tumours, AVM, Masquerader, Robot Excision

## Abstract

An arteriovenous malformation (AVM) is a very rare differential diagnosis of a posterior Mediastinum mass. We report a patient with an AVM mimicking a mediastinal tumour and describe the radiological, pathological findings and the treatment options.

## Introduction

Arteriovenous malformations (AVMs) in the thoracic cavity are extremely rare, with evidence limited to a few case reports. This abnormal communication between arteries and veins can masquerade as infections like tuberculosis, neurogenic tumours or lymphomas.^1^ Rarity, unclear etiology (congenital/traumatic) variable clinical presentation (infectious/ compressive symptoms) and non-specific findings on diagnostic imaging post a diagnostic challenge. We describe a case of a posterior mediastinum AVM in a 20-year-old man, which was treated as tuberculosis till further diagnosis was made.

## Case Presentation

A 20-year-old gentleman, presented elsewhere with complaints of fever along with cough and mucoid expectoration for three weeks and significant loss of weight. A contrast enhanced CT (CECT) scan showed consolidation of the right middle lobe and medial Basal Segment of right lower lobe with a few scattered nodules. In View of typical radiological features (Right middle lobe Syndrome) and strongly positive Mantoux test (sputum negative for AFB), he was started on Anti Tubercular treatment with a 4 drug regime.

**Figure 1:**
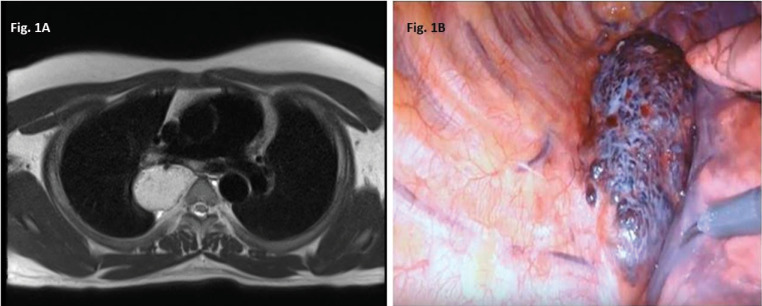
(A) T2 MRI Image showing a hyperintense lesion with closely abutting the azygos vein and maintained fat plane with esophagus and a sharp well demarcated lateral border with the right lung. (B) Intra operative picture of the AVM

He was subsequently referred to our centre and was revaluated. On physical examination, the patient’s chest was clear on percussion and auscultation, but a significant bruit under the right scapular area was noted. A CECT scan demonstrated a non-enhancing posterior mediastinal lesion with few tiny internal calcific specks abutting the vertebral bodies without extension to neural foramina. A MRI revealed an 8x4.6x3.6 cm circumscribed mass lesion with lobulated margin in the right para-vertebral region (D4-D7) suggestive of a neurogenic tumour (Figure 1A). A EUS guided FNAC was inconclusive.

**Figure 2:**
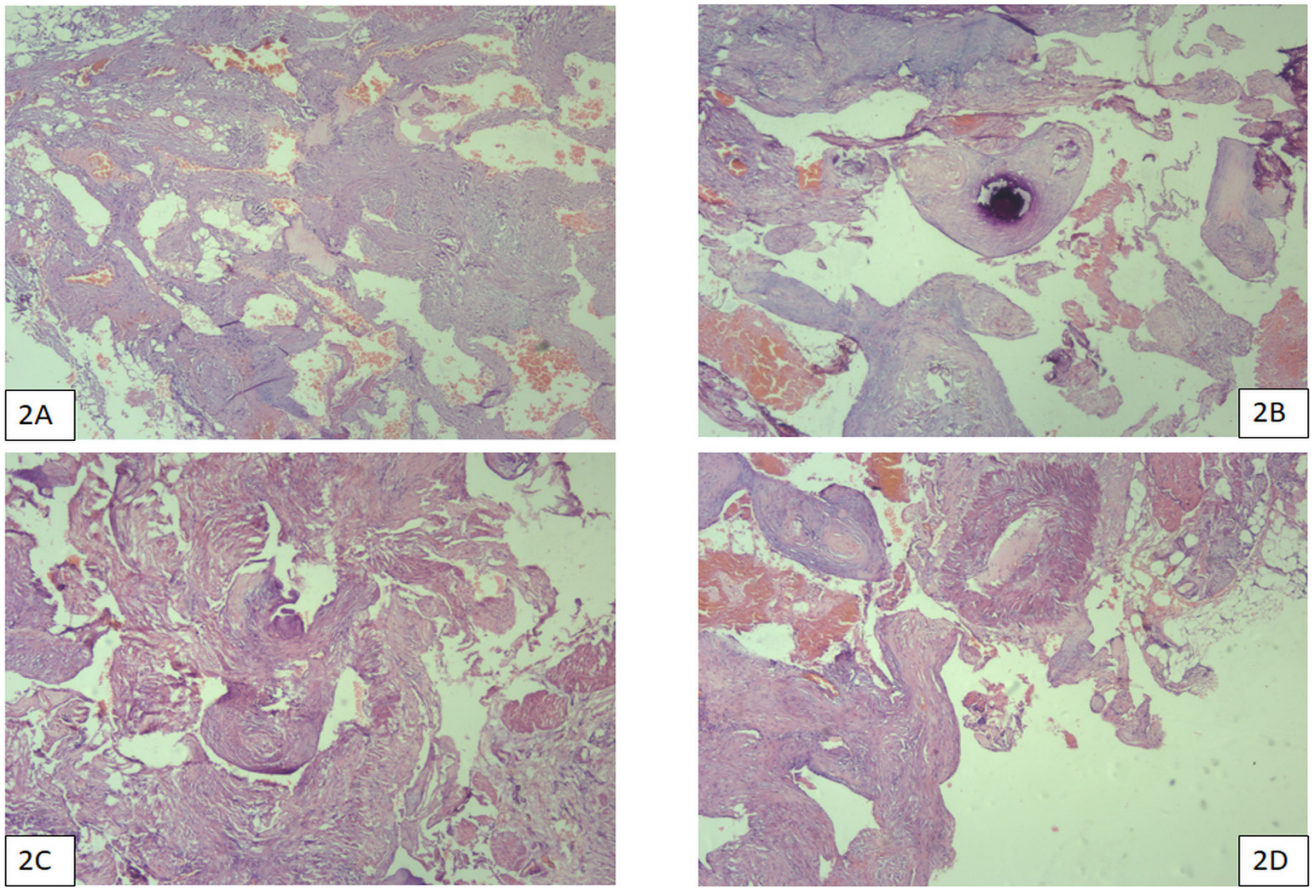
(A, B, C and D) show thick-walled arteries, veins, arterioles and venules of various dimensions with evidence of organized thrombosis (2D) and calcification (2C)

With a presumptive diagnosis of neurogenic tumour of the posterior mediastinum he underwent a robot assisted excision of the posterior mediastinum mass. Intraoperative findings revealed a 6 x 4 cm mass with multiple dilated tortuous veins and 3rd, 4th, 5th right side nerve roots were seen traversing through the mass (Figure 1B). Final histopathology report revealed blood vessels of varying sized calibres with thick walled and thin-walled blood vessels suggestive of an AVM (Figure 2).

## Discussion

Mediastinal AVM’s are a rare entity and are generally large, diffuse and ill-defined involving brachiocephalic, intercostal, and internal mammary arteries.^2^ Presence of a well-defined lesion like in our case is atypical. These lesions can be incidentally detected on chest radiographs or can present with compressive symptoms or in rare scenario heart failure due to the ‘steal phenomenon’. Traumatic Etiology or iatrogenic injury to surrounding vessels is also described.^3^

Differential diagnosis of the lesion includes vascular mediastinal tumors like haemangioma, angiolipoma, and extra lobar pulmonary sequestration or non-vascular lesions like neurogenic tumours. Chronic infections like hydatid cysts and tuberculosis can also be considered in endemic areas.^4^

Surgery with complete excision remains the cornerstone of treatment.^5^ Complete excision can be difficult to the ill-defined nature and encroachment of vital mediastinal structures. Careful planning and preoperative embolization may reduce the intra operative bleeding. In our case we could achieve R0 resection with minimal invasive technique using a robot. We encountered no adhesions or any bleeding during surgery.

## Conclusion

AV malformations in the thorax are uncommon and can be symptomatic at presentation. Lesions that are diagnosed incidentally need to be treated early to avoid further progression. Surgery remains the main treatment of choice.

## Ethical approval

Informed consent was obtained from the patient and the patient identity has been kept confidential in the manuscript.

## Competing interests

None.
